# Revealing parental mosaicism: the hidden answer to the recurrence of apparent de novo variants

**DOI:** 10.1186/s40246-023-00535-y

**Published:** 2023-10-05

**Authors:** Mianne Lee, Adrian C. Y. Lui, Joshua C. K. Chan, Phoenix H. L. Doong, Anna K. Y. Kwong, Christopher C. Y. Mak, Raymond H. W. Li, Anita S. Y. Kan, Brian H. Y. Chung

**Affiliations:** 1 Department of Paediatrics and Adolescent Medicine, School of Clinical Medicine, Li Ka Shing Faculty of Medicine, Queen Mary Hospital, The University of Hong Kong, Room 115, 1/F, New Clinical Building, Pok Fu Lam, Hong Kong SAR China; 2 Department of Obstetrics and Gynaecology, Queen Mary Hospital, Pok Fu Lam, Hong Kong SAR China; 3 Department of Obstetrics and Gynaecology, School of Clinical Medicine, Li Ka Shing Faculty of Medicine, The University of Hong Kong, Pok Fu Lam, Hong Kong SAR China; 4 Prenatal Diagnostic Laboratory, Department of Obstetrics and Gynaecology, Tsan Yuk Hospital, Sai Wan Ho, Hong Kong SAR China; 5 Department of Paediatrics and Adolescent Medicine, Hong Kong Children’s Hospital, Ngau Tau Kok, Hong Kong SAR China

**Keywords:** Parental mosaicism, Gonadal mosaicism, Gonosomal mosaicism, De novo mutation, Recurrence risk, Droplet digital PCR

## Abstract

Mosaicism refers to the presence of two or more populations of genetically distinct cells within an individual, all of which originate from a single zygote. Previous literature estimated the percentage of parental mosaicism ranged from 0.33 to 25.9%. In this study, parents whose children had previously been diagnosed with developmental disorders with an apparent de novo variant were recruited. Peripheral blood, buccal and semen samples were collected from these parents if available for the detection of potential parental mosaicism using droplet digital PCR, complemented with the method of blocker displacement amplification. Among the 20 families being analyzed, we report four families with parental mosaicism (4/20, 20%). Two families have maternal gonosomal mosaicism (*EYA1* and *EBF3*) and one family has paternal gonadal mosaicism (*CHD7*) with a pathogenic/ likely pathogenic variant. One family has a paternal gonosomal mosaicism with a variant of uncertain significance (*FLNC*) with high clinical relevance. The detectable variant allele frequency in our cohort ranged from 8.7–35.9%, limit of detection 0.08–0.16% based on our in-house *EBF3* assay. Detecting parental mosaicism not only informs family with a more accurate recurrence risk, but also facilitates medical teams to create appropriate plans for pregnancy and delivery, offering the most suitable care.

**Supplementary Information:**

The online version contains supplementary material available at 10.1186/s40246-023-00535-y.

## Introduction

Mosaicism refers to the presence of two or more genetically distinct cell populations within an individual, all derived from a single fertilized egg [1]. These cell populations arise from de novo variants (DNVs) that may occur at any developmental stage. Depending on the specific timing of such event, the level and location of mosaicism may be different. Mosaicism can be broadly classified as solely somatic, solely gonadal or gonosomal mosaicism [2]. Mosaicism is commonly found at a variety of levels with variant allele frequencies (VAF) of as low as 0.5% to 3% depending on the detection methods [3, 4]. Traditional Sanger sequencing can only detect mosaicism with a detection limit of 15–20% [5]. The common trio next generation sequencing (NGS) using blood may not be able to distinguish somatic mosaic mutation from gonosomal mosaic mutation, unless multiple sample types such as semen are being tested [6], or inferred from family history due to recurrence of disease. In both gonosomal and gonadal mosaicism, either parent could carry the DNV without any manifestation with the potential to be transmitted to the offspring. It is undetectable or barely detectable in routine genetic tests using blood.

Previous studies estimated the de novo mutation rate for single nucleotide variants (SNVs)/small indels to be approximately 1–1.8 × 10^−8^ per base pair per gamate generation. Therefore, the number of DNVs correlates with parental age at conception, increasing by 1–3 DNVs for every one year increase in age for father and 0.24 DNVs for mother [7]. The disparity primarily stems from the physiological process of spermatogenesis and oogenesis. In females, primordial germ cells (PGCs) arrest in the prophase of meiosis-I and only undergo one additional round of DNA replication during meiosis-II to mature into an ovum. By contrast, male spermatogonial stem cells undergo mitosis every 16 days. Thus, by age 20, a male would have experienced around 190 mitoses, and by age 40, around 660 mitoses. Each replication harbors the potential for incidental copying errors. Hence, approximately 80% of DNVs exhibit what is known as the paternal age effect (PAE) [8]. Although the effect is less severe than paternal age, advanced maternal age also presents a positive correlation with an increased number of apparent DNVs in offspring. Besides, aging oocytes are postulated to accumulate DNVs through alternate mechanism including meiotic gene conversions, crossovers or deficiency in double strand-breaks repair [6, 9].

A specific group of DNVs expand exponentially at significantly higher rates than other DNVs [10, 11]. These highly specific DNVs are observed in genes involved in the RAS-MAPK pathway, which controls cell growth. The mechanism is known as “selfish spermatogonial selection” [10], resulting in a higher risk of sporadic PAE disorders in the offspring of older fathers. Examples of recognized PAE disorders include Apert syndrome (*FGFR2*), Achondroplasia/ Thanatophoric dysplasia (*FGFR3*), Costello syndrome (*HRAS*), Endocrine neoplasia (*RET*) and Noonan syndrome (*PTPN11*) [11]. In addition to the classic PAE genes, there are candidate PAE genes which do not fully satisfy the PAE criteria (e.g., not involved in the RAS pathway) but may still exhibit some PAE features, such as *CHD7* (CHARGE syndrome).

Parental mosaicism increases recurrence risk, and the actual risk depends on the level of parental mosaicism. In some cases, concealed parental mosaicism could be misinterpreted as a DNVs arising in the child. Such misinterpretations could obstruct the accurate estimation of recurrence risk, thereby limiting the options for prenatal or preimplantation genetic testing for families contemplating another child. Despite having a great implication on family planning, particularly for severe diseases with high penetrance and limited medical intervention, current routine practices do not often offer robust detection of low-level parental mosaicism. A recurrence risk of 1% is therefore commonly used when counseling parents whose child is carrying a disease-causing DNV, which may not be always reflecting the truth due to the possibility of hidden low level parental mosaicism that is hindered by current detection limit in terms of sample source and method used.

Previous studies indicated the possibilities of low-level parental mosaicism in transmitting disease-causing variants to the offspring. Although an extensive review by Hancarova et al. [12] reported parental mosaicism from nearly 400 publications in a wide spectrum of diseases, majority of these studies utilized routinely collected samples such as peripheral blood. Zemet et al. [13] in a recent review suggested semen sample may help further stratifying variant with low- or high-risk of recurrence. Indeed, when using semen as the sample type, Bruess et al. [14] in a cohort of patients with Autism Spectrum Disease (ASD) and Frisk et al. [15] in a cohort of patients with intellectual disability (ID) revealed a parental mosaicism diagnostic rate of 21% and 4%, respectively. However, the detection of parental mosaicism using semen samples in other diseases categories remains scarce. Therefore, this study aims to investigate the percentage of parental mosaicism in a cohort of families with apparent DNVs associated with developmental disorders by analyzing the parental blood, semen/or buccal samples with droplet Digital PCR (ddPCR) as the main detection method. We also determined the usefulness of parental mosaicism detection to the genetic counseling and management of patients and their families.

## Methods

### Patient recruitment

**Fig. 1:**
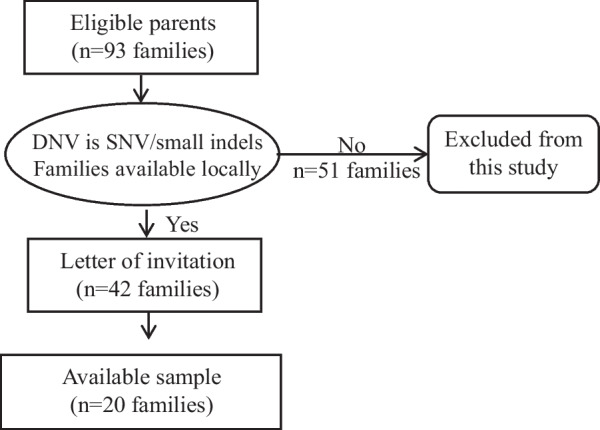
Flowchart for patient recruitment. The diagram shows the recruitment workflow. A total of 20 families are being recruited

Parents of children who had previously received diagnoses of developmental disorders with apparent DNVs identified by trio sequencing using peripheral blood between September 2010 and June 2022 were recruited according to previous sequencing results. There were initially 93 families referred from the clinical genetics service at the Queen Mary Hospital and the Hong Kong Children's Hospital (the University of Hong Kong affiliated hospitals). After that, 42 families were selected according to the following inclusion criteria: i) the apparent DNV is of SNV type or small (< 20 bp) indels, and ii) parents were able to provide freshly obtained buccal and/or semen samples. Invitations were sent to these 42 families, 20 agreed to participate and provided samples. Majority of families (18/20, 90%) were first diagnosed by trio exome sequencing (ES), while 10% (2/20) of families were first diagnosed by trio NGS panel testing. Variant interpretation was based on the American College of Medical Genetics (ACMG) Variant Interpretation Guidelines [16]. The most frequent type of variant is missense (*n* = 10, 50%), followed by nonsense (*n* = 4, 20%), frameshift (*n* = 4, 20%), splice (*n* = 2, 10%), and in frame deletion (*n* = 1, 5%). Three genes were located on chromosome X, while the rest of the genes were located on autosomes. The PAE/candidate PAE genes were *CHD7, COL1A1, RAF1, PTPN11*. The median maternal age at conception was 34.0 years old (range: 25–41) and the median paternal age at conception was 35.5 years old (range: 28 to 44). Semen samples were collected from 18 fathers, and buccal swabs were collected from both mothers (*n* = 8) and fathers (*n* = 17) whenever possible. Written informed consent was obtained from these parents who agreed to participate. This study was approved by the Institutional Review Board of the University of Hong Kong/Hospital Authority Hong Kong West Cluster. (UW 12–211). Figure 1 shows the flowchart for patient recruitment and Additional file 1: table S1 lists the apparent de novo variants for all families in this study.

### Genomic DNA extraction from buccal mucosa and sperm

Genomic DNA was extracted from buccal mucosa within 24 h of collection using Gentra Puregene Buccal Cell Kit (Qiagen, Hilden, Germany) according to the manufacturer’s instruction. Semen samples were also processed within 24 h using the Qiagen QIAamp® DNA Mini Kit (Qiagen, Hilden, Germany) according to the manufacturer’s instruction with overnight incubation to lyse the sperm cells completely. Quantity and quality of the extracted DNA was measured by the Qubit 3.0 Fluorometer (Thermofisher Scientific, Massachusetts, USA) and Nanodrop (Thermofisher Scientific, Massachusetts, USA).

### Sanger sequencing

DNA from buccal mucosa and semen samples was subjected to Sanger sequencing to screen for possible tissue specific mosaic variants. PCR was performed using Qiagen HotStarTaq Plus Master Mix Kit (Qiagen, Hilden,Germany) according to manufacturer instruction. Sanger sequencing was performed at the Center of PanorOmic Sciences (CPOS), The University of Hong Kong.

### Blocker displacement amplification (BDA)/BDA quantitative PCR

BDA was performed for 18 families. Additional file 2: Table S2 lists all the primer pairs and blockers sequences for BDA. Primers to probe ratio were adjusted accordingly case by case during optimization. Parental samples were tested with blocker, while patient samples were tested with blocker and without blocker (with forward and reverse primers only) as the experimental control. Amplified products were Sanger sequenced for verification. BDA-qPCR was performed for Family 14. The reaction mixture contained 10X PowerUp SYBR Green Master Mix (Thermo Fisher Scientific, Waltham, MA), 400 nM of each primer, an optimized amount of blocker and DNA per well. Final volume of 10uL/reaction was loaded on the 7900HT Fast Real-Time PCR System (Applied Biosystems, Foster City, CA). Each reaction was repeated at least twice. Calculations were based on the previous studies by Karolak et al. [17] and Wu et al. [18]. Briefly, change in quantification cycle (∆Cq) values were calculated for each sample using Cq values obtained in both experiments (with and without blocker), i.e., ∆Cq _sample_ = Cq _sample_ (with blocker) − Cq _sample_ (without blocker). VAF were then calculated using the following formula:

$$\frac{50\% }{{2^{{\Delta {\text{Cq}}\;{\text{parent}}}} - \Delta^{{{\text{Cq}}\;{\text{proband}}}} }}$$

### ddPCR technology

A custom TaqMan assay (with primers and probe at 40X concentration) was designed and ordered from ThermoFisher Scientific (Massachusetts, USA) for each individual case (Additional file 3: Table S3), where the reference and variant alleles were labeled as FAM or VIC, respectively. One µl of the above 20 × custom TaqMan assay was mixed with 10 µL of 2 × QX200 ddPCR Probe supermix (no dUTP) (Bio-Rad Laboratories, Munich, Germany), together with 8.25 µl of genomic DNA at ≥ 20 ng/µl and 0.25µL (5U) of digesting enzyme. At least 250 ng of parental samples was used to maximize the chances for low-level mosaicism (< 0.01%) detection as per manufacturer’s guidelines. This 20 µl of ddPCR reaction mixture was then loaded on to the BioRad QX200 ddPCR system according to the manufacturers’ instruction and the analysis was performed as described by Hindson et al [19]. The ddPCR data were analyzed with the QuantaSoft™ analysis software version Pro 1.0.596 (Bio-Rad). Samples with total droplets count of > 8000 were used for subsequent analysis.

## Results

### Percentage of parental mosaicism

Among the 20 families, four families have parental mosaicism (4/20, 20%), in which two families have maternal gonosomal mosaicism (2/20, 10%), one family has paternal gonadal mosaicism (1/20, 5%). The maternal mosaicism in family 17 is a nonsense variant NM_000503.6:c.1081C > T p.(Arg361Ter) in *EYA1*, which has been previously reported [20]. The other maternal mosaic variant is a missense NM_001005463.3:c.488G > A p.(Arg163Gln) variant in the *EBF3* gene. The father in family 14 has a mosaic paternal splice variant in *CHD7* (NM_107780.3:c.7164 + 1G > A). In addition to the three families with parental mosaicism of pathogenic/likely pathogenic variants, the father in family 15 has a mosaic missense variant of uncertain significance (VUS) with high clinical relevance [NM_001458.4:c.4916G > A(p.Cys1639Tyr)] in the *FLNC* gene. Table 1 lists all the pathogenic/likely pathogenic/VUS parental mosaicism included in this study.

**Table 1:** Details of all four families with parental mosaicism (pathogenic/likely pathogenic/variant of uncertain significance) in this study

Family	Gene	Origin	Sample Type	Variant detected by trio ES with peripheral blood				Variant detected by Sanger	Variant detected by BDA/BDA-qPCR		Variant detected by dPCR/ddPCR		Type of Mosaicism
				Reference count	Variant count	Total count	VAF (%)		Enriched	VAF (%)	Total chambers/droplets	VAF (%)	
14	*CHD7*	Maternal	Blood	Unknown (ES by third party)				No	No	Not tested	Not tested		Paternal gonadal mosaicism
			Buccal	Not tested				No	No	Not tested	10,286	< 0.01	
		Paternal	Blood	Unknown (ES by third party)				No	No	Not tested	14,649	< 0.01	
			Buccal	Not tested				No	No	Not tested	14,446	< 0.01	
			Semen	Not tested				Yes	Yes	Not tested	41,644	13.9	
15	*FLNC*	Maternal	Blood	12	0	12	0.0	No	No	< 0.1	16,628	< 0.01	Paternal gonosomal mosaicism
			Buccal	Not tested				No	No	Not tested	17,538	< 0.01	
		Paternal	Blood	8	2	10	20.0	Yes	Yes	12.7	17,905	22.2	
			Buccal	Not tested				Yes	Yes	8.7	36,218	21.3	
			Semen	Not tested				Yes	Yes	22.7	17,510	35.9	
17	^#^*EYA1*	Maternal	Blood	24	4	28	14.3	Yes	Not tested	Not tested	17,284	21.7	Maternal gonosomal mosaicism
		Paternal	Blood	17	0	17	0.0	No			Not tested		
18	*EBF3*	Maternal	Blood	117	17	134	12.7	Yes	Not tested	Not tested	10,203	18.4	Maternal gonosomal mosaicism
			Buccal	Not tested				Yes			12,506	29.6	
		Paternal	Blood	107	0	107	0.0	No			Not tested		

^#^Previously reported family. VAF: Variant allele frequency; BDA: blocker displacement amplification; ES: exome sequencing; qPCR: quantitative polymerase chain reaction; dPCR: digital PCR; ddPCR: droplet dPCR

### Families with parental mosaicism

#### Paternal mosaicism 1: Family 14 with paternal gonosomal mosaicism in *CHD7*

**Fig. 2:**
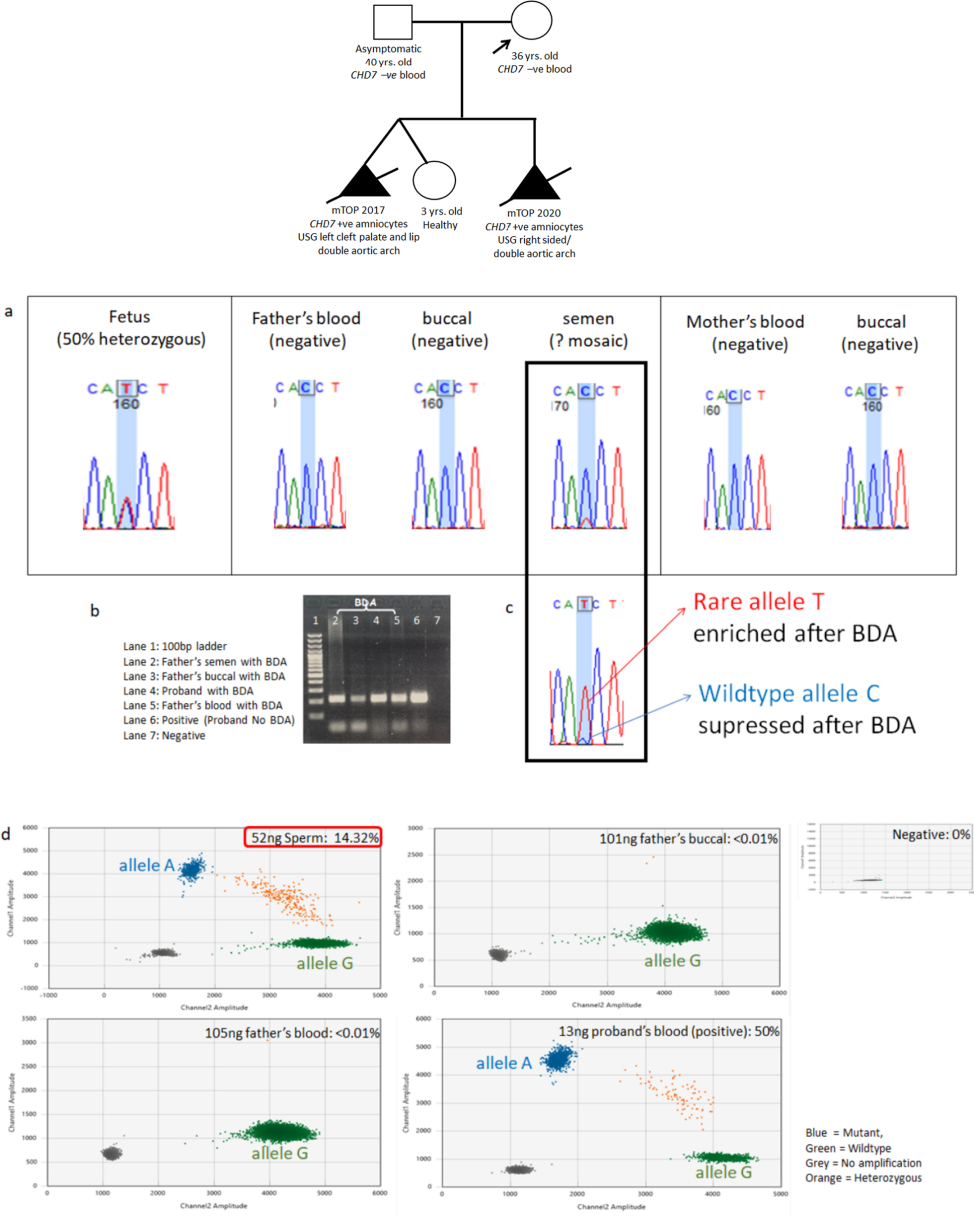
Sanger sequencing and ddPCR results for family 14 with paternal mosaic CHD7 variant. **a** Sanger sequencing showed ambiguous small peak for father’s semen at variant position (black rectangle in bold). **b** Gel electrophoresis with and without BDA. Noted bands with BDA are lighter than that without BDA, demonstrating the suppression of wildtype allele. **c** Sanger sequencing confirmed rare allele T enriched after BDA. **d** DdPCR confirmed paternal gonadal mosaicism with a VAF of 14.32%

Family 14 is previously reported by our team for another RNA study [21] (Fig. 2). Though the variant was not detected in parental blood and buccal cells via Sanger sequencing, a potential mosaic variant was suspected in the father’s semen DNA. With blocker displacement amplification, the rare allele T is enriched and the wildtype allele C is suppressed. DdPCR corroborated the results obtained from Sanger sequencing and BDA, identifying 14.32% of the rare allele in the father’s semen DNA. The detection is highly precise as demonstrated by our three internal serial dilutions with a total input of 52 ng, 26 ng and 13 ng of the same sperm sample, the detectable VAF ranges from 13.6%-14.3% (CV = 0.13 and SD = 0.36).

This confirms the presence of paternal gonadal mosaicism. *CHD7* is a candidate PAE gene with a prevalence of 1/12–15,000 [22]. The detected paternal mosaicism signifies that this family’s risk of recurrence is considerablyhigher than the general prevalence. This is also reflected by the recurrent mutation found in the two affected siblings. Therefore, options such as preimplantation genetic testing for monogenic disease (PGT-M) and prenatal diagnosis for future pregnancy would be beneficial for this family.

#### Paternal mosaicism 2: family 15 with paternal gonosomal mosaicism in *FLNC* (VUS with high clinical relevant)

Family 15 is a non-consanguineous Chinese family. The first child was presented with restrictive cardiomyopathy and a dilated left atrium at 23 months old. He had a deceased younger brother who passed away at 7 months old due to idiopathic restrictive cardiomyopathy. Trio WES using parental blood revealed a VUS in *FLNC* (NM_001458.4:c.4916G > A p.(Cys1639Tyr)), which is suspected to be a paternally inherited mosaic variant due to low coverage with 6 reference reads and two variant reads at 25% VAF*. FLNC* is associated with restrictive cardiomyopathy (MIM: 617,047) which aligns well with the phenotype observed in our patient. The variant is absent in control population (gnomAD v 2.1.1) and software prediction indicates a damaging effect on this variant. Segregation analysis using Sanger sequencing revealed the same variant in the younger brother, which warranted further investigation.

**Fig. 3:**
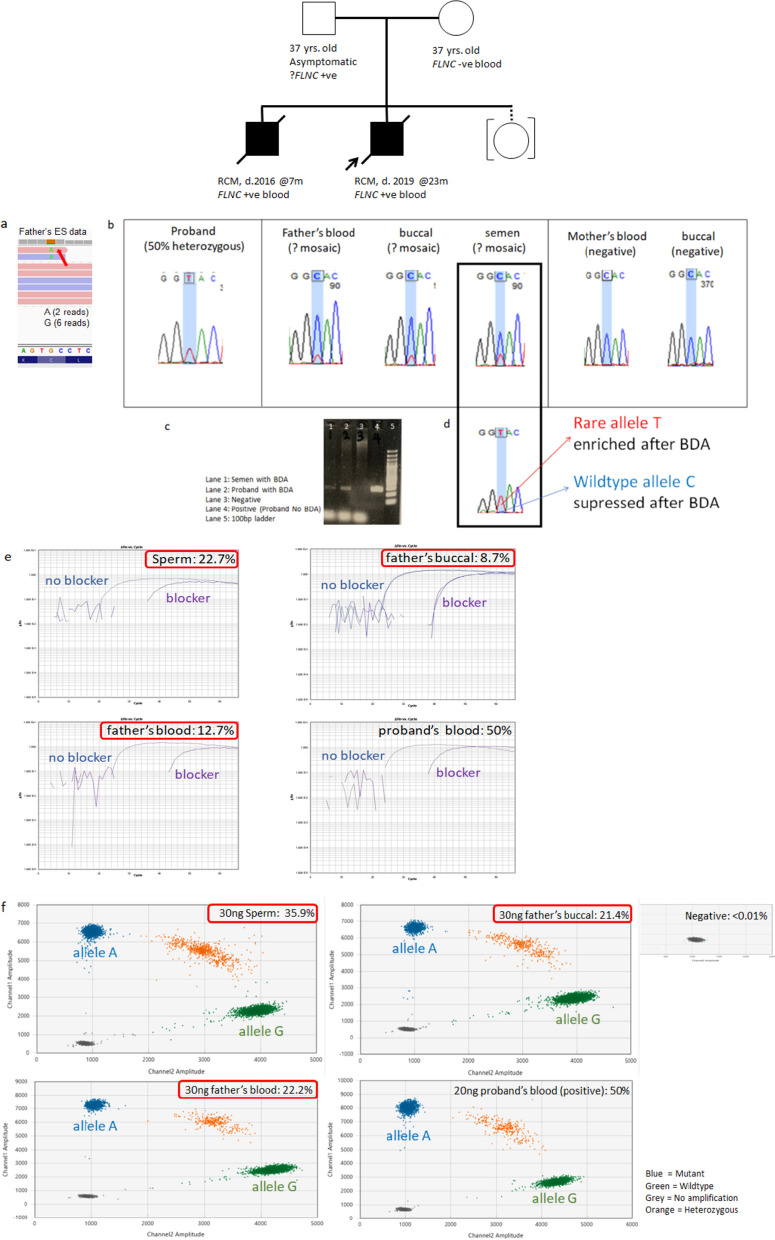
ES, Sanger sequencing, BDA and ddPCR results for family 15 with a paternal mosaic FLNC variant. **a** ES showing low coverage at variant position by. **b** Sanger sequencing showing ambiguous small peak for father’s blood, buccal and semen at variant position (highlighted in blue). **c** Gel electrophoresis with and without BDA. Noted bands with BDA are lighter than that without BDA, demonstrating the suppression of wildtype allele. **d** Sanger sequencing confirmed rare allele T enriched after BDA. **e** BDA-qPCR showing VAF of father’s sperm, buccal and blood at 22.7%, 8.7% and 12.7%, respectively. **f** DdPCR confirmed paternal gonosomal mosaicism with VAF of father’s sperm, buccal and blood at 35.9%, 21.4% and 22.2%, respectively

Using Sanger sequencing, BDA-qPCR and ddPCR, we confirmed an average of 29.3% of the rare allele in the father’s semen DNA, and an average of 17.5% and 15.1% of the rare allele in the father’s blood and buccal sample, respectively, which confirmed paternal gonosomal mosaicism (Fig. 3). Should there be functional evidence of the pathogenicity of the *FLNC* variant, cardiac screening might be necessary for the father. As risk of recurrence may be as high as 29.3%, the option of PGT-M and prenatal diagnosis for future pregnancy would be helpful for the family.

#### Maternal mosaicism: family 18 with maternal gonosomal mosaicism in *EBF3*

**Fig. 4:**
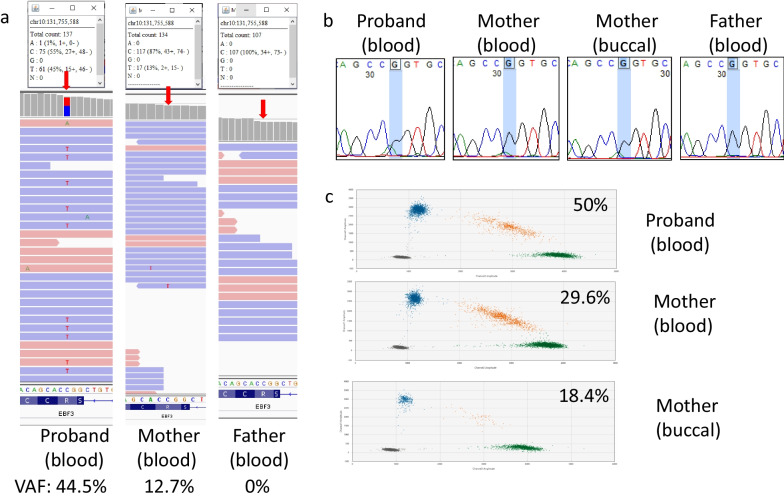
ES, Sanger and ddPCR results for family 18 with maternal mosaic EBF3 variant. **a** IGV showing location of the EBF3 variant in NM_001005463:c.488G > A p.(Arg163Gln) with a red arrow. Maternal mosaicism was initially suspected. **b** Sanger sequencing showed ambiguous small peak for mother at variant position (highlighted in blue). **c** DdPCR confirmed maternal gonosomal mosaicism with 29.6% of the variant allele detected in the buccal sample and 18.4% of the variant allele detected in blood

A 15-year-old Nepalese girl was found to have paraplegia, ataxia and strabismus since birth. She also experienced neurogenic bladder with reflux nephropathy which required intermittent catheterization since age of six. At age 13, she was diagnosed with end stage renal disease and started on hemodialysis. She was referred to our genetic clinic from nephrology due to sudden onset of seizure. Previous trio ESusing peripheral blood revealed a pathogenic heterozygous *EBF3* missense variant in NM_001005463:c.488G > A p.(Arg163Gln), associated with hypotonia, ataxia and delayed development syndrome (MIM: 617,330). The variant is suspected to be inherited from her unaffected mother in a mosaic form as the VAF is skewed at 12.7% with 117 of the reference reads and 17 reads with the variant. Buccal mucosa was then collected for further investigation. Sanger results on both blood and buccal sample showed ambiguous small peak at the variant position, and ddPCR confirmed maternal gonosomal mosaicism with 29.6% of the variant allele detected in buccal and 18.4% of the variant allele detected in blood (Fig. 4). The maternal mosaicism indicated that an increase in risk of recurrence for this family, so, PGT-M and prenatal diagnosis is useful for this family.

In-house ddPCR limit of detection (LOD) was determined using the *EBF3* assay, in which the detected VAF were comparable with the expected VAF when VAF was expected to be 0.08–0.16% (Figure S2). Theoretically LOD should be systemically determined on per assay based; however, with limited resources and control DNA, *EBF3* assay was used and results were comparable with manufacturer’s guideline and other similar studies [19, 23, 24].

## Discussion

This study investigated parental mosaicism in 20 families, in which the children had been previously diagnosed with developmental disorders with apparent de novo variants identified through trio sequencing. Utilizing family history, additional biological samples including buccal mucosa and semen samples, coupled with sensitive technologies including ddPCR and BDA, we illustrated that 20% (4/20) of the families have parental mosaic variants which is previously regarded as DNVs in the patients.

### Cohort characteristics

The median maternal age at conception in our cohort was 34.0 years, ranging from 25–41 years of age. Excluding the three families without an affected first child, the median maternal age at first childbirth for our cohort was 34.4 years old (ranging from 26–40 years old), slightly higher than the data reported by the Census and Statistics Department Hong Kong in 2021 [25], where the maternal median age at first childbirth was 32.6 years. The median paternal age at first childbirth for our cohort is 36.0 years old (ranging from 29–44 years old). There was a lack of Census data for paternal median age at first childbirth for a similar comparison, highlighting that discussions regarding parental age at childbirth tend to focus predominantly on females, despite the significant role of paternal age [22]. According to Census data [25], both the median age at first marriage for female and male has been steadily increasing, from 27.5 and 30.2, respectively, in 2001 to 30.6 and 32.2, respectively, in 2021, which can be inferred that maternal and paternal age at first childbirth may also be on a steady increase. While our current societal focus revolves around maternal age as a public health concern, it is equally important to educate the public about the potential risk of advanced paternal age and the burdens it may bring.

### Percentage of parental mosaicism and clinical implications

Despite the significant differences in inclusion criteria, detection methods and sample types used, the diagnostic yield of 4/20 (20%) disease-causing parental mosaicism found in our cohort with diverse Mendelian diseases was comparable with previous studies, where parental mosaicism were found between 0.3%-26.5% [4, 26, 27] in cohorts that focused on similar diverse Mendelian disease. Among the families with detected parental mosaicism, half had two affected siblings. This suggests that parental mosaicism should be strongly considered in families where the disease recurs. Moreover, our data suggest that the empirical VAF obtained from laboratory tests may further help predict recurrence risk. A higher VAF in multiple tissues indicates an earlier occurrence during embryonic development leading to a higher recurrence risk. This is exemplified in family15 with paternal gonosomal mosaicism, in which both affected offspring inherited the *FLNC* variant from the father. We identified the paternal mosaic variant in tissues representing ectoderm (buccal), intermediate mesoderm (sperm) and mesoderm (blood). The average VAF in sperm was higher (29.3%) compared to blood and buccal samples (average VAF of 14.5% and 15.0%, respectively). These results imply that low level parental mosaicism is prone to be missed if only blood or buccal samples are tested.

The mutational event in family 14 with the paternal gonadal *CHD7* variant would have occurred later after differentiation of PGCs since the mosaic variant is confined in the semen at 13.9% and not found in blood and buccal. The recurrence risk in this family appears to be smaller than that of family 15, as demonstrated by the presence of two affected and one healthy offspring. However, as *CHD7* is a candidate PAE gene associated with proliferation advantages; the recurrence risk may increase with the father’s age. Although Pauli et al. [28] did not find any PAE in their cohort of affected children carrying *CHD7* variants of paternal origin (*n* = 12), earlier studies by Tellier et al [29] (*n* = 41) and Blake et al [30] (*n* = 39) suggested an association between *CHD7* mutation and PAE.

Although PAE may increase the prevalence of paternal mosaicism, and previous studies report a ratio of paternal to maternal DNVs is at 4:1 [8, 31, 32], our observations confirm that maternal mosaicism still exist, as demonstrated by two families in our study. To increase the sample size, we reviewed parental mosaicism studies with more than ten families since 2009 (Table 2). Among those with a known positive parental mosaic variant affecting non-sex chromosome, the incidence of paternal mosaicism and maternal mosaicism were comparable at 59% (47/80) and 41% (33/80), respectively. On the other hand, X-linked recessive diseases (e.g., *DMD*) have a higher maternal mosaicism rate and it is not surprising because males are more likely to have an X-linked recessive diseases as their X-chromosome can only be inherited from their mother. Besides, X-linked dominant diseases seems to show a higher rate of paternal mosaicism (e.g., *MECP2* and *PCDH19* in Rett Syndrome and Developmental and epileptic encephalopathy 9, respectively, as shown in Table 2), however, a more thorough literature search that includes all cases using an unbiased approach is required to confirm these preliminary findings.

**Table 2:** Literature review on parental mosaicism since 2009 for cohort studies with more than 10 families

Disease	Author	Year	Sample Size	Sample Type	Detection Method	Parental Mosaicism					
						Paternal	Maternal	Unknown	Total	Percentage	Detectable %VAF
AHC	Yang et al	2019	80 families	Blood/Saliva/Buccal mucosa/Hair/Skin/Urine/Sperm	Deep sequencing/SS/mddPCR	4	2	0	6/80	7.5%	0.03–33.03%
Autism	Krupp et al	2017	2264 families	Blood	ES	63*	49*	0	NA	6.80%	7.9–36.1%
	Breuss et al	2020	14 families	Sperm	GS	3	0	0	3/14	21.43%	0.6–14.5%
Developmental and epileptic encephalopathy	Liu et al	2019	22 families	Blood/Buccal mucosa/Hair/Nails/Urine	Targeted sequencing/SS/MLPA/mddPCR	2	0	0	2/22	9.09%	1.2–-37.38%
Diverse Mendelian Disease	Cao et al	2019	12,000 samples (120 candidate parental mosaic variants)	Blood	ES/Deep sequencing/Sanger Sequencing	14	25	1	40/12,000	0.33%	3.1–67.8%
	Gambin et al	2020	2000 families (102 candidate parental mosaic variant)	Blood/Saliva/Buccal mucosa/Hair/Urine	ES/Deep sequencing/ddPCR/BDA	0	0	27	27/102	26.47%	0.3–18.2%
	Shu et al	2021	237 families	Blood/Saliva/Buccal mucosa/Hair/Urine	Deep sequencing/ddPCR	4	10	0	NA	3.00%	0.22–34.0%
	This study	2022	21 families	Blood/Buccal mucosa/Sperm	ES/SS/BDA/ddPCR	1	2	1	4/21	19.00%	8.7–35.9%
DMD	Helderman-van den Enden et al	2009	318 families	Blood	Haplotyping	0	19	0	19/318	5.97%	NA
	Zhong et al	2019	74 families	Blood	Targeted sequencing/MLPA	0	2	0	2/74	2.70%	NA
Epilepsy	Depienne et al	2010	177 families	Blood/Sperm	SS,QAS-PCR, haplotyping	6	7	0	13/177	7.34%	0.04–24%
	Xu et al	2015	174 families	Blood/Saliva/Buccal mucosa/Hair/Urine	Deep sequencing/SS/MLPA/dPCR	13	7	0	20/174	11.49%	1.1–32.6%
	Yang et al	2017	112 families	Blood/Sperm	Deep sequencing/SS /MLPA/mddPCR	18	11	0	29/112	25.89%	0.01–39.04%
	Myers et al	2018	120 families	Blood/Saliva	smMIP	6	4	0	10/120	8.33%	1.4–3.6%
	de Lange et al	2019	80 families	Blood	Deep sequencing/ddPCR	3	1	0	4/80	5.00%	0.5–8.0%
	Rikke S Møller	2019	75 families	Blood/Buccal mucosa/Urine	Gene panel sequencing	4	1	0	5/75	6.67%	0.8–29%
Holoprosencephaly	Paulussen et al	2010	86 families	Blood/Sperm	SS/haplotyping	0	1	0	1/86	1.16%	NA
	Hu et al	2019	136 families	Blood	Targeted sequencing/ddPCR	2	3	0	5/136	3.68%	0.1–13%
ID	Acuna-Hidalgo et al	2017	50 families	Blood	GS	3	1	0	4/50	8.00%	0.22–6.15%
	Wright et al	2019	420 families	Blood/Saliva	ES/Deep sequencing	13	8	0	21/420	0.50%	0.5–33.0%
	Frisk et al	2022	44 families	Blood/Sperm	ES/ddPCR	2	0	0	2/44	4.55%	1.1–20.24%
Malformation of cortical development	Zillhardt et al	2016	18 families	Blood	ES/ddPCR/SS	1	2	1	4/18	22.22%	4.31–4.57%
Mandibulofacial Dysostosis with Microcephaly	Huang et al	2016	94 families	Blood	Sanger Sequencing/Haplotyping	0	1	0	1/94	1.06%	NA
Marfan and Ehlers-Danlos syndromes	Chesneau et al	2021	333 families	Blood	Targeted sequencing/SS/HRMA	2	1	0	3/62	4.84%	1.1–13.6%
MPSII	Alcantara-Ortigoza et al	2016	25 families	Blood//Buccal mucosa/Hair/Urine	SS	0	1	0	1/25	4.00%	NA
OI	Pyott et al	2011	37 families	Blood	SS	2	4	0	6/37	16.22%	NA
	Shaheen et al	2012	13 families	Blood	SS	0	0	2	2/13	15.38%	NA
PID	Mensa-Vilaro et al	2019	92 families	Blood//Buccal mucosa/Hair/Urine/Sperm	Gene panel/SS	1	5	1	7/92	7.61%	2.7–21.2%
Rett syndrome	Zhang et al	2018	21 families	Blood/Saliva/Sperm	ddPCR	5	0	0	5/21	23.81%	0.03–7.55%
X-linked ALD	Wang et al	2011	489 families	Blood	SS	1	2	1	4/489	0.82%	NA

AHC: Alternating hemiplegia of childhood; ALD: adrenoleukodystrophy; ES: exome sequencing; VAF: Variant allele frequency; BDA: blocker displacement amplification; DMD: Duchenne muscular dystrophy; dPCR: digital polymerase chain reaction; ddPCR: droplet dPCR; HRMA: high-resolution melting analysis; mddPCR: micro ddPCR; MLPA: Multiplex Ligation-dependent Probe Amplification; ID: intellectual disability; MPSII: Mucopolysaccharidosis type II; OI: Osteogenesis imperfecta; PID: Primary immunodeficiency diseases; SS: Sanger Sequencing. *Numbers extracted from best practice filter

Indeed, the most accurate method for detecting gonadal mosaicism should involve direct observation of germ cells. While sperm can be sued to detect paternal gonadal mosaicism, maternal gonadal mosaicism would require an invasive biopsy of ovaries [33], which is impractical in most cases. Therefore, known cases of maternal gonadal mosaicism are likely to be underestimated. Notably for the same reason, the absence of detectable mosaicism in paternal semen does not necessarily stratify low recurrence risk unless the DNV of interest is known to phase to paternal allele, which usually requires long-read sequencing that is not commonly accessible in routine clinical laboratories [14]. Caution is required to completely rule out maternal mosaicism. Nonetheless, the use of blood and saliva analysis could possibly pick up both maternal and paternal gonosomal mosaicism [34].

### Comparison with previous studies

Although the first few reported parental mosaicism cases in the late 1980s and early 1990s mainly focused on isolated families with genetic disorders such as Duchenne muscular dystrophy and osteogenesis imperfecta [35, 36] with limited molecular evidence available. However, the rapid advancement of molecular technologies, particularly NGS, has accelerated the identification ofparental mosaicism in a broader range of disorders, for example epilepsy [37–39], ASD [14, 40, 41] and developmental delay (DD) [3], as well as a wide spectrum of genetic disorders including Marfan [42], Noonan [43], polycystic kidney [44], primary immunodeficiency diseases [45] and congenital heart diseases [46] (Table 2).Earlier studies, such as Myers et al. [37] showed that among 120 children having epilepsy with an apparently DNV, approximately 10% of them had a parent with mosaicism. Krupp et al. [41] also showed that parental mosaicism was found to range from 7–11% in a large ASD cohort with 2300 families using blood as the sample type. Using semen as the sample type, Breuss et al. [14] showed that 29% (4/14) fathers were mosaic for the causative DNVs transmitted to their ASD-affected children. For intellectual disability (ID) caused by DNVs, paternal mosaicism was found in 4.7–6.5% of the families in cohorts of around 50 patients [7, 15]. In a cohort of 237 patients with a DNVs among a wide spectrum of developmental disorders, Shu et al. [27] also found 3% parental mosaicism using ES as the detection method at read depth of 2000X.

Based on the above studies, it can be concluded that parental mosaicism can be found in 3–29% of various developmental disease cohorts. Diagnostic yield for parental mosaicism is much lower at 0.3–0.5% if a non- targeted approach was used, as demonstrated in two large cohort studies using ES at a read depth of 50-130X as the initial detection method. Wright et al. [3] examined trio ES data of 4,293 probands at ~ 50 X average depths from the Deciphering Developmental Disorders (DDD) Study and only 0.5% of parental mosaicism was found. Similar study performed by Cao et al. [4] based on ES data of 11,992 probands at 130X average depth from Baylor Genetics identified only 0.3% parental mosaicism in the analyzed families. While only seven mosaic variants were identified directly by trio ES, 33 mosaic variants could be found during Sanger confirmation. These larger cohort studies with > 4000 patients indicated that parental mosaicism may have been underestimated unless a more targeted approached is implemented in clinical settings. Despite its increasingly strong association with many disorders and great clinical impact, detection of mosaicism is still mostly passive and technically challenging. This underscores the need for continued research and development of more sensitive and targeted detection methods.

### Technical advice

Our study, in line with previous studies, had demonstrated that the detection of parental mosaicism is challenging as mosaicism maybe tissue-specific or tissue-limited [2, 3, 12, 14, 20, 26, 47]. Using Sanger sequencing as a golden standard and blood as the most clinically accessible sample type may miss the detection of low- level parental mosaicism. While isolated study demonstrated that LOD of Sanger sequencing could go as low as 0.25% [48], such LOD require thorough optimization of the whole Sanger sequencing procedure, and depends on the particular location of the mosaic variants. BDA-qPCR is a valuable tool in determining low-level mosaicism although the experimental conditions need to be extensively optimized and technically more demanding than Sanger sequencing and ddPCR. The robustness coupled with high sensitivity and precision of ddPCR provides a reliable alternative for the detection of low-level parental mosaicism. Based on the *EBF3* assay, in-house ddPCR LOD is determined to be 0.08%-0.16%, which is comparable to manufacturer’s guideline (Figure S2). Therefore, our data demonstrated the feasibility of routine clinical analysis for parental mosaicism evaluation in families in need, using buccal and/or semen samples. With the increased accessibility and affordability of NGS technology, high depth sequencing may also help reveal more cases of parental mosaicism [3, 14]. While GS is not yet offered as the first-tier genetic testing in routine clinical setting, with increased throughput and lower costs, universal GS-based clinical genetic testing is within reach. Once this becomes routine, the chances of detecting these low-level mosaic variants through massive parallel sequencing at the current 30X read depth might diminish. Recent advancements in bioinformatics such as DeepMosaic [49] may have the potential to reveal mosaic SNVs with GS at 50X; however, an orthogonal method may still be needed to validate these potential mosaic findings [50]. Consequently, we recommend careful examination of the potential for parental mosaicism in instances where an apparent de novo variant has been identified through either routine exome sequencing or genome sequencing, particularly for families contemplating a second child. It is crucial for diagnostic laboratories to thoroughly validate their methodologies, including assessment of sensitivity, precision, and accuracy, to ensure reliable findings that can effectively inform both healthcare professionals and parents."

In conclusion, the approach to diagnosing low-level parental mosaicism should start at the genetic clinic. Attention should be given to cautious observation of any mild symptom in parents, an increased paternal age at conception or a previously affected child with PAE/candidate PAE or X-linked dominant disorders. Although paternal mosaicism is known to be common, possibility of maternal gonadal mosaicism cannot be excluded. Technically, the detection of parental mosaicism relies on using appropriate sample types, such as buccal and/or semen samples, in conjunction with sensitive methods that exceed those routinely applied in clinical diagnostics. The robust detection of parental mosaicism is crucial as it permits accurate assessment of recurrence risk during genetic counseling. This information allows families to make early, informed decisions about future pregnancies. Additionally, it enables prenatal medical teams to formulate appropriate plans for pregnancy, prenatal testing, and delivery.

### Supplementary Information

**Additional file 1**. Apparent de novo variants for all families in this study.

**Additional file 2**. Primer and probe sequences for the BDA experiments.

**Additional file 3**. Primer and probe sequences for the ddPCR experiments.

## Data Availability

The datasets used and/or analyzed during the current study are available from the corresponding author on reasonable request.
