# Supplementary material for: Revealing parental mosaicism: the hidden answer to the recurrence of apparent de novo variants
Source: Hum Genomics. 2023 Oct 5;17:91. doi: 10.1186/s40246-023-00535-y (PMC10557286; doi:10.1186/s40246-023-00535-y)
Supplement: Supplementary file 1 — Additional file 1. Apparent de novo variants for all families in this study. [file 40246_2023_535_MOESM1_ESM.docx]

Table S1. Apparent *de novo* variants for all families in this study.

| **Family** | **Gene** | **Family History^#^** | **DNV** | **ACMG classification** | **PAE/**  **Candidate PAE genes** |
| --- | --- | --- | --- | --- | --- |
|  |  |  |  |  |  |
|  |  |  |  |  |  |
| 7 | *ARID1A* | No | c.3406G>A p.(Ala1136Thr) | Pathogenic | No |
| 9 | *ARID1B* | No | c.5394_5397delTGTT p.(Phe1798LeufsTer52) | Pathogenic | No |
| 19 | *ARID1B* | No | c.3136-2A>G | Pathogenic | No |
| 14 | *CHD7* | More than 1 affected child | c.7164+1G>A | Pathogenic | Yes |
| 5 | *COL1A1* | No | c.1426G>A p.(Gly476Arg) | Likely pathogenic | Yes |
| 12 | *DDX3X* | 2 abortions | c.1462del p.(Arg488fs) | Pathogenic | No |
| 18 | *EBF3* | No | c.488G>A p.(Arg163Gln) | Likely pathogenic | No |
| 17 | *EYA1* | No | c.1081C>T p.(Arg361Ter) | Pathogenic | No |
| 15 | *FLNC* | More than 1 affected child | c.4916G>A p.(Cys1639Tyr) | VUS | No |
| 10 | *FOXG1* | No | c.321_345del p.(Pro108Tspfs*76) | Pathogenic | No |
| 13 | *KDM6A* | No | c.1083_1084delCT p.(Tyr362*) | Pathogenic | No |
| 4 | *PPP1CB* | No | c.548A>C p.(Glu183Ala) | Pathogenic | No |
| 20 | *PPP1R12A* | No | c.609del p.(His204ThrfsTer12) | Pathogenic | No |
| 16 | *PTEN* | No | c.278A>G p.(His93Arg) | Pathogenic | No |
| 2 | *PTPN11* | G4P1, History of miscarriage | c.5C>T p.(Thr2Ile) | Pathogenic | Yes |
| 1 | *RAF1* | Father with oligosemenia, history of testicular cancer | c.788T>G p.(Val263Gly) | Likely Pathogenic | Yes |
| 3 | *ROBO* | No | c.4122G>A p.(Trp1374*) | Likely pathogenic | No |
| 8 | *SCN2A* | No | c.4972C>T p.(Pro1658Ser) | Pathogenic | No |
| 6 | *SRP54* | No | c.349_351del p.(Thr117del) | Pathogenic | No |
| 11 | *WDR45* | No | c.249G>A p.(Trp83*) | Pathogenic | No |

ACMG: American College of Medical Genetics; DNV: *de novo* variant; PAE: paternal age effect; VUS: variant of uncertain significance. ^#^Family history relevant to mosaicism is defined as those who had 1) recurrent of disease in the family, 2) threatened abortion or miscarriage 3) history of testicular cancer
