# Supplementary material for: Revealing parental mosaicism: the hidden answer to the recurrence of apparent de novo variants
Source: Hum Genomics. 2023 Oct 5;17:91. doi: 10.1186/s40246-023-00535-y (PMC10557286; doi:10.1186/s40246-023-00535-y)
Supplement: Supplementary file 2 — Additional file 2. Primer and probe sequences for the BDA experiments. [file 40246_2023_535_MOESM2_ESM.docx]

Table S2. Primer and probe sequences for the BDA experiment.

| **Family** | **Gene** | **Oligonucleotide** | **Nucleotide sequence (5' -> 3')** |
| --- | --- | --- | --- |
| 1 | *RAF1* | Forward Primer | ACAGGCAGGGTGGTGCTGA |
|  |  | Reverse Primer | CAGGTAGAGTTTGCCCTTAAGCA |
|  |  | Blocker | GGTGCTGACCATGTGGACATTAGGTGTGGTAG/3SpC3/ |
| 2 | *PTPN11* | Forward Primer | AGGGCGGGAGGAACATGA |
|  |  | Reverse Primer | TCGGGGAGGCAGGAAATGAA |
|  |  | Blocker | CGGGAGGAACATGACATCGCGGAGGTGAGG/3dSp/ |
| 3 | *ROBO1* | Forward Primer | TTGTCCTCCTCTGAGGCTGA |
|  |  | Reverse Primer | AGAAGACGAAGCCGACATGG |
|  |  | Blocker | TCTGAGGCTGAGCCCCAGCCGTTGATCATGG/3SpC3/ |
| 4 | *PPP1CB* | Forward Primer | GTCACCAGACCTGCAATCTA |
|  |  | Reverse Primer | GTAATCTGTAGCTGGCTTATTGAC |
|  |  | Blocker | CCAGACCTGCAATCTATGGAGCAGATTCGGAG/3SpC3/ |
| 5 | *COL1A1* | Forward Primer | TCCGGGCAGGCCAGT |
|  |  | Reverse Primer | CCAGAAAGGAAGAGGAGCCC |
|  |  | Blocker | GGCCAGTGGGTCCGGGTTCACCT/3SpC3/ |
| 6 | *SRP54* | Forward Primer | GCAAGGGAGTGGTAAAACAACA |
|  |  | Reverse Primer | CAAACCTATCTTCCATATGACTTCT |
|  |  | Blocker | GGGAGTGGTAAAACAACAACATGTTCAAAGG/3SpC3/ |
| 7 | *ARID1A* | Forward Primer | CCAAGATCCAGCCTCCCTCT |
|  |  | Reverse Primer | AAGAGCTGCCGTAGAGTACC |
|  |  | Blocker | GCCTCCCTCTCCTGGTAAGGATGGGGTC/3InvdT/ |
| 8 | *SCN2A* | Forward Primer | CTACAGGAATGTTTCTGGCTGAACT |
|  |  | Reverse Primer | TGAGCTTCCAGGGTGATCTTTG |
|  |  | Blocker | GCTTTGATGATGTCCCTTCCTGCGTTGTTTAAC/3Phos/ |
| 9 | *ARID1B* | Forward Primer | AAGCACTTGATCACAACGCAG |
|  |  | Reverse Primer | TCCATCTTGCTCTCAAAGTGAGT |
|  |  | Blocker | GAACAACCTGTTTGTTGTTGACCGATCTG/3Phos/ |
| 10 | *FOXG1* | Forward Primer | AGCAGCTGCTGCTCCC |
|  |  | Reverse Primer | TGCTTGTTCTCGCGGTAGTA |
|  |  | Blocker | TGCTCCCGCCGCCGCCACCGCCA/3SpC3/ |
| 11 | *WDR45* | Forward Primer | TTGCCCTCCCGGGCAT |
|  |  | Reverse Primer | CGGAAATCTCCAGGGTGCAT |
|  |  | Blocker | CGGGCATCGTCCCAGATCAGCACTGCT/3SpC3/ |
| 12 | *DDX3X* | Forward Primer | AGAGGGATAGAGAAGAGGCC |
|  |  | Reverse Primer | TTACCAAGGTTTCCTACACGTCC |
|  |  | Blocker | GAAGAGGCCCTTCACCAGTTCCGCTCAGGAAA/3InvdT3/ |
| 13 | *KDM6A* | Forward Primer | GCAGCCTGGATGGACCT |
|  |  | Reverse Primer | AAATACTTAATTCGTGCTGCAAGT |
|  |  | Blocker | GGATGGACCTAGGCACTCTCTATGAATCCTGC/3SpC3/ |
| 14 | *CHD7* | Forward Primer | CGTCTCCTCAGTTGTCAAAG |
|  |  | Reverse Primer | CTGCTATATGGGCTGGCTTT |
|  |  | Blocker | CCTCAGTTGTCAAAGGTGAATTAGAATGGCT/3SpC3/ |
| 15 | *FLNC* | Forward Primer | ACTGGGGATGCCAGCAAGT |
|  |  | Reverse Primer | GCCCAATCTGGATTCGAGGG |
|  |  | Blocker | GATGCCAGCAAGTGCCTCGTCACAGGTGGG/3SpC3/ |
| 16 | *PTEN* | Forward Primer | GCACAATATCCTTTTGAAGACC |
|  |  | Reverse Primer | GTGCCTTTAAAAATTTGCCCCG |
|  |  | Blocker | CACAATATCCTTTTGAAGACCATAACCCACCACAGC/3SpC3/ |
| 17 | *EYA1* | Forward Primer | Not tested |
|  |  | Reverse Primer |  |
|  |  | Blocker |  |
| 18 | *EBF3* | Forward Primer | CCACAACTTTTCTTGTCACAGCAC |
|  |  | Reverse Primer | AAGTCGGGCATAAAAGGGGGAAAT |
|  |  | Blocker | CTTTTCTTGTCACAGCACCGGCTGTGGAGCAATTG/3SpC3/ |
| 19 | *ARID1B* | Forward Primer | CCACATGCTGCTTCTGGGTAC |
|  |  | Reverse Primer | CAGACTTGAGACAGGAGAGCCT |
|  |  | Blocker | GCTGCTTCTGGGTACTAGAAGTCCAGCTCCTCCACC/3SpC3/ |
| 20 | *PPP1R12A* | Forward Primer | AGGCCAAAAGGTCACAGAGA |
|  |  | Reverse Primer | CTTTAGCAGCTGCAACGT |
|  |  | Blocker | GCAGCTGCAACGTGAAGTGCTGTACCTCCA/3SpC3/ |
