# Supplementary material for: Revealing parental mosaicism: the hidden answer to the recurrence of apparent de novo variants
Source: Hum Genomics. 2023 Oct 5;17:91. doi: 10.1186/s40246-023-00535-y (PMC10557286; doi:10.1186/s40246-023-00535-y)
Supplement: Supplementary file 3 — Additional file 3. Primer and probe sequences for the ddPCR experiments. [file 40246_2023_535_MOESM3_ESM.docx]

Table S3. Primer and probe sequences for the ddPCR experiments.

| **Family ID** | **Gene** | **Oligonucleotide** | **Nucleotide sequence (5' -> 3')** | **Amplicon (5' -> 3')** |
| --- | --- | --- | --- | --- |
| 1 | *RAF1* | Forward Primer | AGGCAGGGTGGTGCTG | AGGCAGGGTGGTGCTGACCATGTGG[A/C]CATTAGGTGTGGATGTCGACCTCTG |
|  |  | Reverse Primer | AGGCAGAGGTCGACATCCA |  |
|  |  | FAM Probe | ACCATGTGGCCATTAG |  |
|  |  | VIC probe | ACCATGTGGACATTAG |  |
| 2 | *PTPN11* | Forward Primer | Undisclosed sequence from Thermofisher (Assay ID: C_322101298_10) | GAGCCGGAGGGCGGGAGGAACATGA[C/T]ATCGCGGAGGTGAGGAGCCCCGAGG |
|  |  | Reverse Primer |  |  |
|  |  | FAM Probe |  |  |
|  |  | VIC probe |  |  |
| 3 | *ROBO1* | Forward Primer | CTGGAAATGTTGTCCTCCTCTGA | TGTTGTCCTCCTCTGAGGCTGAGCC[C/T]CAGCCGTTGATCATGGACCCCGTGA |
|  |  | Reverse Primer | GGGACCTGGAGAGCTCTGT |  |
|  |  | FAM Probe | CTGAGCCTCAGCCGT |  |
|  |  | VIC probe | CTGAGCCCCAGCCGT |  |
| 4 | *PPP1CB* | Forward Primer | GGATTGTCACCAGACCTGCAA | TTGTCACCAGACCTGCAATCTATGG[A/C]GCAGATTCGGAGAATTATGAGACCT |
|  |  | Reverse Primer | GTATCAGGGACATCAGTAGGTCTCA |  |
|  |  | FAM Probe | CGAATCTGCGCCATAG |  |
|  |  | VIC probe | CTCCGAATCTGCTCCATAG |  |
| 5 | *COL1A1* | Forward Primer | CGGGCAGGGACACTTACAC | GGGGGTCCGGGCAGGCCAGTGGGTC[C/T]GGGTTCACCTCGAGCTCCTCGCTTT |
|  |  | Reverse Primer | TGCTGGAGAGGAAGGAAAGC |  |
|  |  | FAM Probe | TGAACCCAGACCCAC |  |
|  |  | VIC probe | TGAACCCGGACCCAC |  |
| 6 | *SRP54* | Forward Primer | GATTATGTTTGTTGGATTGCAAGGGA | TTGCAAGGGAGTGGTAAAACAACA[*/ACA]TGTTCAAAGGTAAATTGAACTTAA |
|  |  | Reverse Primer | TGATTTATACAAACCTATCTTCCATATGACTTCTTTTT |  |
|  |  | FAM Probe | AAAACAACAACATGTTCAAA |  |
|  |  | VIC probe | TGGTAAAACAACATGTTCAAA |  |
| 7 | *ARID1A* | Forward Primer | AGTCCCAGCCCAAGATCCA | GCCCAAGATCCAGCCTCCCTCTCCT[G/A]GTAAGGATGGGGTCAGCGGCCCCAC |
|  |  | Reverse Primer | GCCCTCTCAGCCTTGGT |  |
|  |  | FAM Probe | CCTCCCTCTCCTAGTAAG |  |
|  |  | VIC probe | CCTCCCTCTCCTGGTAAG |  |
| 8 | *SCN2A* | Forward Primer | CGCTGCTCTTTGCTTTGATGA | GCTCTTTGCTTTGATGATGTCCCTT[C/T]CTGCGTTGTTTAACATCGGCCTCCT |
|  |  | Reverse Primer | GAAAAGAAGGAGGCCGATGTTAAAC |  |
|  |  | FAM Probe | AACGCAGAAAGGGACA |  |
|  |  | VIC probe | AACGCAGGAAGGGACA |  |
| 9 | *ARID1B* | Forward Primer | AGTTCGACAAGCTGCCAATAAAGAT | AAAGATAGTCAAAAAGAACAACC[TGTT/*]TGTTGTTGACCGATCTGACAAGTT |
|  |  | Reverse Primer | ACGCCCCAACTTGTCAGAT |  |
|  |  | FAM Probe | TCAACAACAGGTTGTTC |  |
|  |  | VIC probe | ACAACAAACAGGTTGTTC |  |
| 10 | *FOXG1* | Forward Primer | CGCCGACGACGACAAG | ACAAGGGCCCCCAGCAGCTGCTGCTCCCGCCGCC[GCCACCGCCACCACCGGCCGCCGCC/*]CTGGACGGGGCTAAAGCGGACGGGCTGGGCGG |
|  |  | Reverse Primer | AGCCCGTCCGCTTTAGC |  |
|  |  | FAM Probe | CTCCCGCCGCCCTGG |  |
|  |  | VIC probe | CCACCACCGGCCGCC |  |
| 11 | *WDR45* | Forward Primer | CAGCTTCTCCTTGGAGTCCTT | AGTCCTTGCCCTCCCGGGCATCGTC[C/T]CAGATCAGCACTGCTGGGCAGGTGG |
|  |  | Reverse Primer | ACCTTGGCCCCGACAAC |  |
|  |  | FAM Probe | TGCTGATCTGAGACGAT |  |
|  |  | VIC probe | TGCTGATCTGGGACGAT |  |
| 12 | *DDX3X* | Forward Primer | AGACCGTTCTCAGAGGGATAGAG | TAGAGAAGAGGCCCTTCACCAGTTC[C/*]GCTCAGGAAAAAGCCCAATTTTAGT |
|  |  | Reverse Primer | CATACTGCTGTAGCCACTAAAATTGG |  |
|  |  | FAM Probe | TTTTCCTGAGCGAACTG |  |
|  |  | VIC probe | TCCTGAGCGGAACTG |  |
| 13 | *KDM6A* | Forward Primer | CCATGCTGCAGCCTGGAT | CTGCAGCCTGGATGGACCTAGGCA[CT/*]CTCTATGAATCCTGCAACCAGCCTC |
|  |  | Reverse Primer | CTGAGGCTGGTTGCAGGATT |  |
|  |  | FAM Probe | CCTAGGCACTCTATG |  |
|  |  | VIC probe | ACCTAGGCACTCTCTATG |  |
| 14 | *CHD7* | Forward Primer | GCATTAGTGGGAGTGAGGACATC | CACTACGTCTCCTCAGTTGTCAAAG[G/A]TGAATTAGAATGGCTTGTTTCTGCA |
|  |  | Reverse Primer | TTTAAGCTGCAGAAACAAGCCATTC |  |
|  |  | FAM Probe | CAGTTGTCAAAGATGAATT |  |
|  |  | VIC probe | CAGTTGTCAAAGGTGAATT |  |
| 15 | *FLNC* | Forward Primer | CCCTTCCGCATCCATGCT | CTGCCCACTGGGGATGCCAGCAAGT[G/A]CCTCGTCACAGGTGGGTGCCCAC |
|  |  | Reverse Primer | GTGGGCACCCACCTGT |  |
|  |  | FAM Probe | CCAGCAAGTACCTCGTC |  |
|  |  | VIC probe | CAGCAAGTGCCTCGTC |  |
| 16 | *PTEN* | Forward Primer | GGTTATCTTTTTACCACAGTTGCACAA | GTTGCACAATATCCTTTTGAAGACC[A/G]TAACCCACCACAGCTAGAACTTATC |
|  |  | Reverse Primer | TGGTCAAGATCTTCACAAAAGGGTTT |  |
|  |  | FAM Probe | TTGAAGACCGTAACCC |  |
|  |  | VIC probe | TTGAAGACCATAACCC |  |
| 17 | *EYA1* | Forward Primer | Undisclosed sequence from Thermofisher (Assay ID: C_173826997_10) | AAGTTGAAAATCATTTCTTCCATTC[G/A]CAGTCCAAGGGAAACTGAAGTGGGT |
|  |  | Reverse Primer |  |  |
|  |  | FAM Probe |  |  |
|  |  | VIC probe |  |  |
| 18 | *EBF3* | Forward Primer | CGTTTCGTTTCTATTGCCACAACTT | GCCACAACTTTTCTTGTCACAGCAC[C/A]GGCTGTGGAGCAATTGTAAACAGTG |
|  |  | Reverse Primer | GGGAAATGCATATTAAAACCACTGTTTACA |  |
|  |  | FAM Probe | TCCACAGCCTGTGCTG |  |
|  |  | VIC probe | CACAGCCGGTGCTG |  |
| 19 | *ARID1B* | Forward Primer | CCACCTCCACATGCTGCTT | CCTCCACATGCTGCTTCTGGGTACT[A/G]GAAGTCCAGCTCCTCCACCACTACT |
|  |  | Reverse Primer | CGTGATCTTCTCCCCAGTAGTG |  |
|  |  | FAM Probe | TGGGTACTGGAAGTC |  |
|  |  | VIC probe | CTGGGTACTAGAAGTC |  |
| 20 | *PPP1R12A* | Forward Primer | CTTCCGTATAGCCTTTAGCAGCT | TATAGCCTTTAGCAGCTGCAACGTG[A/*]AGTGCTGTACCTCCAGATTTTGCAT |
|  |  | Reverse Primer | CGGCATGCAAAATCTGGAGGTA |  |
|  |  | FAM Probe | CAGCACTCACGTTGC |  |
|  |  | VIC probe | CAGCACTTCACGTTGC |  |

FAM: a fluorescent dye with an absorption wavelength of 495 nm and an emission wavelength of 517 nm (Carboxyfluorescein); VIC: a fluorescent dye with an excitation peak at 526 nm and an emission peak at 543 nm.
